# Mendelian Randomization Study: The Association Between Metabolic Pathways and Colorectal Cancer Risk

**DOI:** 10.3389/fonc.2020.01005

**Published:** 2020-07-23

**Authors:** Su Yon Jung, Jeanette C. Papp, Eric M. Sobel, Zuo-Feng Zhang

**Affiliations:** ^1^Translational Sciences Section, Jonsson Comprehensive Cancer Center, School of Nursing, University of California, Los Angeles, Los Angeles, CA, United States; ^2^Department of Human Genetics, David Geffen School of Medicine, University of California, Los Angeles, Los Angeles, CA, United States; ^3^Department of Epidemiology, Fielding School of Public Health, University of California, Los Angeles, Los Angeles, CA, United States; ^4^Center for Human Nutrition, David Geffen School of Medicine, University of California, Los Angeles, Los Angeles, CA, United States

**Keywords:** Mendelian randomization, genetically driven insulin resistance, obesity, physical activity, high-fat diet, colorectal cancer, postmenopausal women

## Abstract

**Background:** The roles of obesity-related biomarkers and their molecular pathways in the development of postmenopausal colorectal cancer (CRC) have been inconclusive. We examined insulin resistance (IR) as a major hormonal pathway mediating the association between obesity and CRC risk in a Mendelian randomization (MR) framework.

**Methods:** We performed MR analysis using individual-level data of 11,078 non-Hispanic white postmenopausal women from our earlier genome-wide association study. We identified four independent single-nucleotide polymorphisms associated with fasting glucose (FG), three with fasting insulin (FI), and six with homeostatic model assessment–IR (HOMA-IR), which were not associated with obesity. We estimated hazard ratios (HRs) for CRC by adjusting for potential confounding factors plus genetic principal components.

**Results:** Overall, we observed no direct association between combined 13 IR genetic instruments and CRC risk (HR = 0.96, 95% confidence interval [CI]: 0.78–1.17). In phenotypic analysis, genetically raised HOMA-IR exhibited its effects on the increased risk and FG and FI on the reduced risk for CRC, but with a lack of statistical power. Subgroup analyses by physical activity level and dietary fat intake with combined phenotypes showed that genetically determined IR was associated with reduced CRC risk in both physical activity-stratified (single contributor: *MTRR* rs722025; HR = 0.12, 95% CI: 0.02–0.62) and high-fat diet subgroups (main contributor: *G6PC2* rs560887; HR = 0.59, 95% CI: 0.37–0.94).

**Conclusions:** Complex evidence was observed for a potential causal association between IR and CRC risk. Our findings may provide an additional value of intervention trials to lower IR and reduce CRC risk.

## Introduction

Postmenopausal women have a high incidence of and mortality from colorectal cancer (CRC). Approximately 90% of newly diagnosed CRC patients and CRC-related deaths occur in women aged 50 years and older (1); thus, their high risk for CRC development and worse prognosis contribute to CRC's third place rank in cancer incidence and mortality among women of the United States and other Westernized counties (2, 3). While obesity (both overall and abdominal) is a well-known risk factor (4, 5), the roles of obesity-related biomarkers and their molecular pathways connected to colorectal carcinogenesis have not been conclusive. In particular, the insulin resistance (IR)/insulin-like growth factor-I (IGF-I) axis has been considered a major hormonal pathway and has played a crucial mediating role in the association between obesity and CRC development (5). For example, *in vitro* and *in vivo* studies showed that insulin stimulates the growth of colorectal tumors in cell lines (6) and animal models (7). Additionally, metformin, a medicine used to regulate glucose homeostasis, suppresses the activity of obesity-related CRC cells (8).

Molecular biologic studies suggest potential mechanisms for the association between IR and CRC risk, including overexpression of insulin and IGF-I receptors (9–11) and hyperregulation and dysregulation of downstream cell-signaling pathways (10, 12–14), leading to the reduced apoptotic and enhanced anabolic cellular state necessary for tumor growth and development. However, previous clinical and observational epidemiologic studies for the relationship between IR and CRC risk among women ages 50 years and older are inconsistent. Some studies have found blood glucose levels and homeostatic model assessment–IR (HOMA-IR) associated with CRC (15, 16) while other studies have found no associations of glucose, insulin, and HOMA-IR levels with CRC (17, 18). In particular, one study examining postmenopausal women revealed that fasting glucose levels were associated with increased CRC risk (16), while another study (18) using the same study population showed no clear association between HOMA-IR and CRC risk. Those inconsistent findings may be in part due to selection bias, confounding, and/or modifying effects of obesity and associated lifestyle factors, relatively short-time exposure to biomarkers, and reverse causation; further, the lack of consensus in these findings calls for in-depth research such as Mendelian randomization (MR) studies, which could potentially improve the causal inference.

An MR approach could address those challenges. It examines genetic variants (e.g., single-nucleotide polymorphisms [SNPs]) as an instrumental variable to evaluate the effect of the genetically determined exposure (e.g., IR) on an outcome (e.g., CRC risk) (19). This genetic analysis may be a useful tool to establish a relatively unbiased causal relationship between IR and CRC risk by circumventing potential biases and residual confounding. Since random assortment of alleles occurs when a gamete is formed, this can lead to random assignment of exposure (19, 20). In addition, an MR approach may examine a lifelong exposure to an allele because the random assignment of genetic variations occurs at meiosis (20). Furthermore, MR prevents reverse causation in that the randomly assigned genetic variations precede the phenotypes and clinical outcomes (20, 21).

In the current study, we performed MR analysis by using our earlier genome-wide association study (GWAS) results (22) to test the hypothesis that genetically determined IR has a potential causal association with CRC risk in postmenopausal women.

## Materials and Methods

### Data Sources and Selection of Candidate Instrumental Variables

We analyzed the data from our earlier meta-analysis of a genome-wide gene–environmental (i.e., behavioral) interaction (G × E) study (22) among 11,078 non-Hispanic white women after menopause. Those women were enrolled in the Women's Health Initiative (WHI) Harmonized and Imputed GWASs under the WHI Database for Genotypes and Phenotypes (dbGaP) Study, accession number phs000200.v11.p3. The detailed study rationale and design have been described elsewhere (23, 24). Briefly, healthy postmenopausal women were enrolled in the WHI study between 1993 and 1998 from more than 40 clinical centers across the United States. They were eligible for the WHI dbGaP study if they had met eligibility requirements for submission to dbGaP and provided DNA samples. The WHI Harmonized and Imputed studies consist of six GWASs. From the six GWASs, we obtained the genotyped data. The genotyped calls were normalized to reference panel GRCh37, and genotype imputation was performed using the 1,000 Genomes Project reference panel (24). In the initial and secondary data-quality cleaning processes, we included SNPs with a missing-call rate of <3%, a Hardy–Weinberg equilibrium of *p* ≥ 10^−4^, and an imputation quality of ${\hat{R}}^{2}\geq 0.6$, in our previous G × E GWAS meta-analysis. The study was approved by the institutional review boards of each participating clinical center of the WHI and the University of California, Los Angeles.

We used the results from our meta-analysis of the G × E GWAS for IR and CRC risk. We identified IR-associated SNPs at genome-wide significance (*p* <5 × 10^−8^) and used them as genetic instrumental variables in this study. We further pruned SNPs according to linkage disequilibrium (LD) estimates to select SNPs with *r*^2^ <0.1. Among the 58 SNPs associated with IR phenotypes overall or in subgroups stratified by obesity, physical activity level, and high-fat diet intake, we ultimately identified the following independent SNPs: (1) four independent SNPs associated with fasting glucose (FG) levels (one in the overall, two in the physically active, and one in the high-fat diet groups); (2) three associated with fasting insulin (FI) levels (one in the obese, one in the physically inactive, and one in the low-fat diet groups); and (3) six associated with HOMA-IR (two in the overall, two in the low-fat diet, and two in the high-fat diet groups).

### Statistical Analysis

For each identified SNP, we conducted multiple Cox proportional hazards regression analyses to obtain the hazard ratios (HRs) and 95% confidence intervals (CIs) for CRC risk by checking assumptions with a Schoenfeld residual plot and rho. The analyses were adjusted for potential confounding factors. The confounding factors were selected through literature review (1, 25) for their associations with IR and CRC risk and from the initial analysis process, including univariate and stepwise multiple regression analyses and a multi-collinearity test: 10 genetic principal components (PCs) as well as age, education, family income, depressive symptoms, cardiovascular disease ever, hypertension ever, high cholesterol, family history of CRC, physical activity, smoking, height, body mass index (BMI), waist-to-hip ratio, dietary alcohol, dietary fiber, daily fruits, daily vegetables, percentage of calories from saturated fatty acids (SFA), monounsaturated fatty acids, polyunsaturated fatty acids, and protein, hysterectomy ever, ages at menarche and menopause, breastfeeding, oral contraceptive duration, and exogenous estrogen (E)-only and E plus progestin use.

We first checked basic MR assumptions to see whether our data fulfilled the conditions required for valid causal inference. Traditionally in MR analysis, genetic instruments should not have a weak relationship with their phenotypes. To address that, we estimated a sum of the *T*-squared statistics across SNPs for the overall and specified groups by phenotype and by subgroup (Table S1). By using the commonly used threshold of 10 units (26), we found that our SNP instruments were well powered for downstream MR analysis.

In addition, an MR approach could be confounded when the analyzed SNPs present biological pleiotropy or are independently associated with CRC risk through intermediate pathways other than IR. To assess whether our data has a potential pleiotropic effect, we utilized the following analytic approaches. Considering that obesity is a well-established risk factor for CRC and could exhibit its pleiotropic effect independently of or interrelatedly with the IR-CRC pathway, we interrogated for the association of obesity (27) with the modeled SNPs to exclude from the MR analysis. No evidence of SNPs having pleiotropic association with obesity was observed. We further conducted an MR-Egger analysis (28) and tested for directional pleiotropy, which indicates that the pleiotropic effect across SNPs is skewed on outcome in one direction rather than being balanced. No significant directional pleiotropic effect of SNPs by phenotype and by subgroup was found (Table S2).

Having found that our SNPs have sufficient strength to predict relevant phenotypes and are less likely to be confounded, we next performed MR analysis by employing the inverse-variance weighted method (29). This quantifies the association between genetically derived IR and CRC risk. During the MR analysis, we took into account a population correlation that could occur when exposure (IR) and outcome (CRC development) were assessed within the same population. We therefore adjusted for Spearman correlation rho between each IR phenotype and CRC in the analysis. For the individual genetic-instrumental effects on the risk for CRC, we estimated the ratio of β coefficients (=ß_colorectalcancer_/ß_IR_) (20). The results were reported as risk ratios (RRs) and 95% CIs and interpreted as the change in CRC risk per unit increase in log-odds of IR or the change in RR (exponentiation of β) for women with IR compared with women without.

To test for additional evidence of pleiotropy, we examined the heterogeneity of MR estimates by using Cochran's *Q*-test. We considered a two-tailed *p* <0.05 statistically significant. We used R statistical software (v 3.5.1).

## Results

The 13 IR SNPs in the different subgroups from our earlier G × E GWAS and their risk for CRC development are presented in Table 1. In particular, the two SNPs *PABPC1P2* rs77772624 and *LINC00460* rs17254590, in relation to HOMA-IR, were shown at genome-wide association in the overall and high-fat diet (calories from SFA ≥ 7%) subgroups.

**Table 1:** Characteristics of IR SNPs and their effect on IR and CRC risk.

**Gene**	**SNP**	**Chr**	**Position**	**Allele**	**Alternative allele frequency**		**IR**			**CRC risk**	
				**Ref/Alt**	**Controls**	**CRC**	**OR**	***P***	**Q**	**HR£ (95% CI)**	***P***
					**(*n* = 10,342)**	**(*n* = 736)**					
**Fasting glucose**											
G6PC2^*^	**rs13431652**	**2**	**169,753,415**	**T/C**	0.30	0.32	**0.79**	**6.99E-09**	**0.706**	1.07 (0.96–1.19)	0.244
*G6PC2*^§^	**rs560887**	**2**	**169,763,148**	**T/C**	0.29	0.32	**1.28**	**6.12E-09**	**0.513**	**0.88** (**0.78**–**0.99**)	**0.027**
*MKLN1*^†^	**rs117911989**	**7**	**130,969,793**	**G/A**	0.05	0.03	**1.98**	**3.97E-08**	**0.209**	**0.56 (0.34**–**0.91)**	**0.020**
*NKX2-2*^†^	**rs7273292**	**20**	**21,473,362**	**T/C**	0.01	0.0001	**3.37**	**4.35E-08**	**0.148**	1.09 (0.54–2.18)	0.813
**Fasting insulin**											
*NR5A2*^¶^	**rs10919774**	**1**	**199,907,716**	**G/A**	0.95	0.95	**1.98**	**2.53E-08**	**0.726**	0.87 (0.57–1.34)	0.531
*MTRR/LOC729506*^€^	**rs722025**	**5**	**8,108,012**	**G/A**	0.75	0.73	**1.28**	**3.73E-08**	**0.643**	**0.59 (0.40**–**0.89)**	**0.011**
*PLA2G4A*^¥^	**rs6683451**	**1**	**187,292,608**	**A/C**	0.11	0.09	**3.16**	**4.86E-08**	**0.230**	1.88 (0.24–14.95)	0.552
**HOMA-IR**											
PABPC1P2^*^	**rs77772624**	**2**	**147,499,474**	**A/C**	0.001	0.001	**29.65**	**4.96E-09**	**0.634**	1.01 (0.25–4.09)	0.987
*PABPC1P2*^§^	**rs77772624**	**2**	**147,499,474**	**A/C**	0.001	0.001	**28.92**	**9.36E-09**	**0.711**	0.54 (0.08–3.84)	0.535
*MSC*^¥^	**rs13277245**	**8**	**72,606,942**	**A/G**	0.18	0.18	**29.57**	**4.92E-08**	**N/A**	1.37 (0.83–2.27)	0.224
*DOCK1*^¥^	**rs113847670**	**10**	**128,874,679**	**C/T**	0.04	0.03	**9.18**	**2.85E-08**	**0.571**	0.41 (0.11–1.60)	0.201
LINC00460^*^	**rs17254590**	**13**	**107,037,344**	**G/C**	0.02	0.0004	**2.52**	**2.40E-08**	**0.620**	0.64 (0.09–4.69)	0.661
*LINC00460*^§^	**rs17254590**	**13**	**107,037,344**	**G/C**	0.02	0.0004	**2.67**	**8.86E-09**	**0.882**	0.66 (0.09–4.87)	0.685

*Alt, alternative allele; Chr, chromosome; CI, confidence interval; CRC, colorectal cancer; HOMA-IR, homeostatic model assessment–insulin resistance; HR, hazard ratio; IR, insulin resistance; N/A, not available; OR, odds ratio; Q, Cochran's Q; Ref, reference allele; SNP, single–nucleotide polymorphism. Numbers in bold face are statistically significant*.

*£ HR was estimated by adjusting for confounding factors (10 genetic principal components plus age, education, family income, depressive symptoms, cardiovascular disease ever, hypertension ever, high cholesterol, family history of CRC, physical activity, smoking, height, body mass index, waist-to-hip ratio, dietary alcohol, dietary fiber, daily fruits, daily vegetables, % calories from saturated fatty acids, monounsaturated fatty acids, polyunsaturated fatty acids, and protein, hysterectomy ever, ages at menarche and at menopause, breastfeeding, oral contraceptive duration, and exogenous estrogen [E]-only, and E plus progestin use)*.

* *SNPs at genome-wide level identified in the overall analysis*.

§ *SNPs at genome-wide level in subgroup analysis within the high-fat diet group (≥7.0% calories from saturated fatty acids [SFA])*.

† *SNPs at genome-wide level in subgroup analysis within the physically active group (≥10 metabolic equivalent [MET])*.

¶ *SNPs at genome-wide level in subgroup analysis within the obese group (body mass index ≥ 30.0 kg/m^2^)*.

€ *SNPs at genome-wide level in subgroup analysis within the physically inactive group (<10 MET)*.

¥ *SNPs at genome-wide level in subgroup analysis within the low-fat diet group (<7.0% calories from SFA)*.

**Figure 1:**
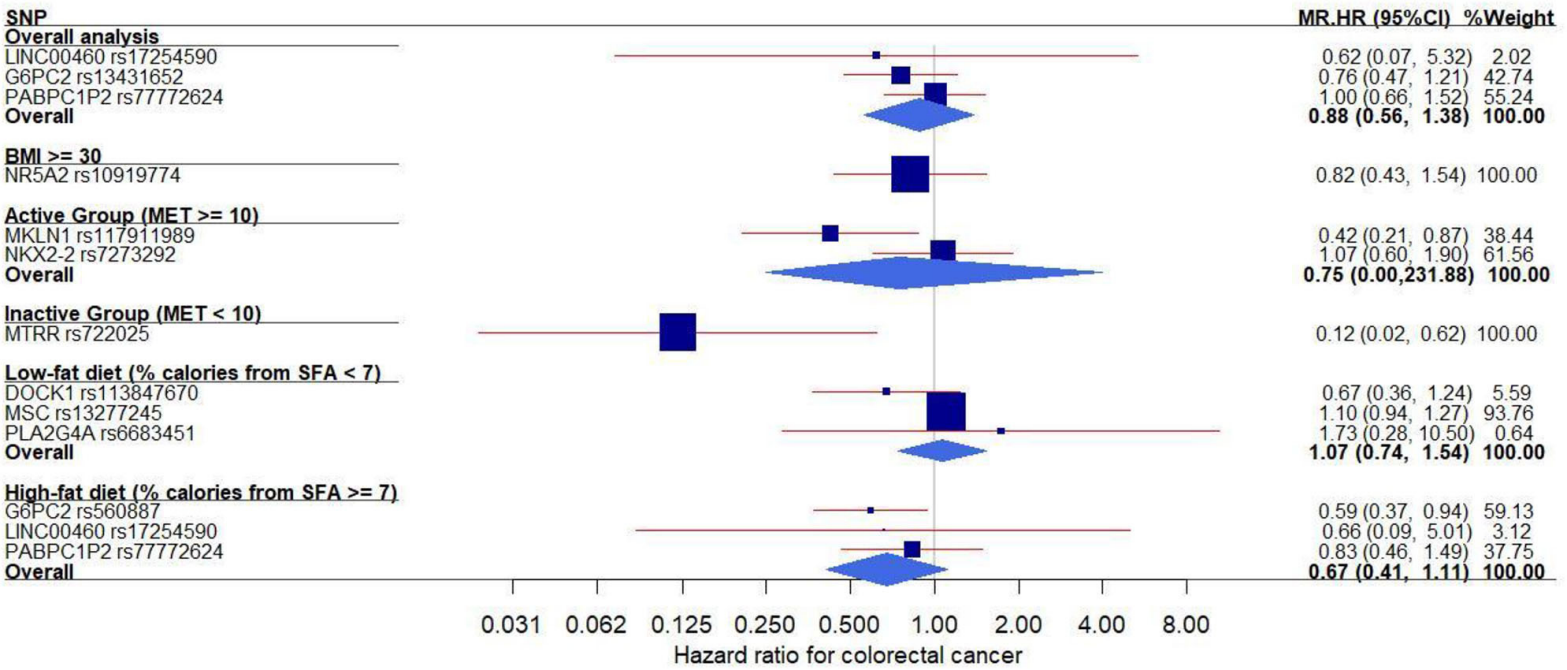
Forest plot of the MR effects of IR on colorectal cancer risk in the overall and subgroups. For each of non-pleiotropic IR SNPs, the plot shows the effects of genetically driven IR (FG, FI, or HOMA-IR) on colorectal cancer risk in the overall and subgroups, presented as the 95% CIs (indicated with red lines) of the estimates and the inverse-variance weights (percentages proportional to the size of the blue squares). BMI, body mass index; CI, confidence interval; FG, fasting glucose; FI, fasting insulin; HOMA-IR, homeostatic model assessment–insulin resistance; HR, hazard ratio; IR, insulin resistance; MET, metabolic equivalent; MR, Mendelian randomization; SFA, saturated fatty acids; SNP, single–nucleotide polymorphism.

In the MR analysis of individual genetic instruments for the association between their respective phenotype (FG, FI, and HOMA-IR) and CRC risk, we identified three SNPs whose genetically determined effect of IR on CRC risk was statistically significant (Table 2 and Figure 1). The SNPs *G6PC* rs560887 (FG, HR = 0.59, 95% CI: 0.37–0.94) in the high-fat diet subgroup; *MKLN1* rs117911989 (FG, HR = 0.42, 95% CI: 0.21–0.87) in the physically active (metabolic equivalent [MET] ≥ 10) subgroup, and *MTRR* rs722025 (FI, HR = 0.12, 95% CI: 0.02–0.62) in the physically inactive (MET <10) subgroup were associated with CRC risk. When the genetic instruments were combined by phenotype and evaluated for CRC risk (Table 2), the pooled estimates of genetically derived FG and FI were associated with reduced CRC risk whereas that of genetically derived HOMA-IR was associated with slightly increased CRC risk, although those estimates were not statistically significant.

**Table 2:** Mendelian randomization analysis for the effect of IR on colorectal cancer risk.

**Subgroup**	**Fasting glucose**			**SNP**	**Fasting insulin**			**SNP**	**HOMA-IR**			**SNP**
	HR (95% CI)^¶^^*^	***P***	***P*_**hat**_**	***n***	HR (95% CI)^¶^^*^	***P***	***P*_**hat**_**	***n***	**HR (95% CI)^¶^**	***P***	***P*_**hat**_**	***n***
Overall	0.76 (0.47–1.21)	0.244	N/A	1					0.99 (0.32–3.07)	0.903	0.664	2^€^
BMI ≥ 30					0.82 (0.43–1.54)	0.531	N/A	1				
Active (MET ≥ 10)	0.75 (0.002–232.37)	0.638	0.050	2^†^								
Inactive (MET <10)					**0.12 (0.02–0.62)**	**0.011**	**N/A**	1				
% calories from SFA <7.0					1.73 (0.28–10.50)	0.552	N/A	1	1.07 (0.26–4.35)	0.655	0.125	2^¥^
% calories from SFA ≥ 7.0	**0.59 (0.37–0.94)**	**0.027**	**N/A**	1					0.82 (0.37–1.81)	0.191	0.826	2^§^
Pooled estimate	0.70 (0.41–1.19)	0.120	0.207	4	0.70 (0.09–5.52)	0.538	0.060	3	1.06 (0.87–1.30)	0.429	0.442	6

*BMI, body mass index; CI, confidence interval; HOMA-IR, homeostatic model assessment–insulin resistance; HR, hazard ratio; IR, insulin resistance; MET, metabolic equivalent; SFA, saturated fatty acids; SNP, single–nucleotide polymorphism. Numbers in bold face are statistically significant*.

*P_hat_ was estimated on the basis of Cochran's Q*.

¶ *The Mendelian randomization HR was estimated by adjusting for correlation rho between each phenotype and colorectal cancer risk within the same population*.

* *The Mendelian randomization effects of single SNPs on colorectal cancer risk were estimated via the ratio of ß-coefficients (=ß_colorectalcancer_/ß_IR_) (20)*.

€ *Two SNPs were PABPC1P2 rs77772624 and LINC00460 rs17254590*.

† *Two SNPs were MKLN1 rs117911989 and NKX2-2 rs7273292*.

¥ *Two SNPs were MSC rs13277245 and DOCK1 rs113847670*.

§ *Two SNPs were PABPC1P2 rs77772624 and LINC00460 rs17254590*.

**Figure 2:**
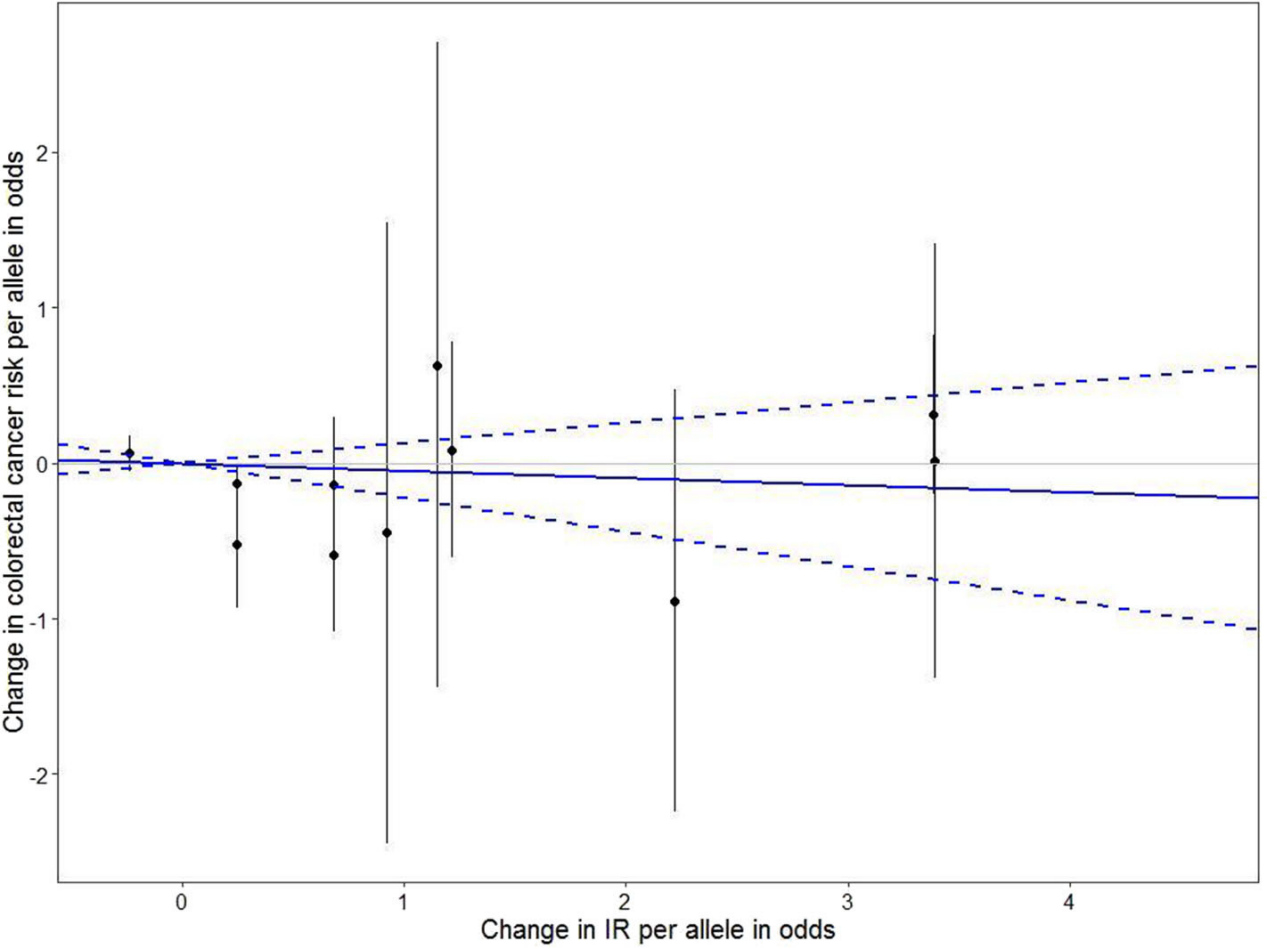
The effect of individual IR-genetic instrumental variables on colorectal cancer risk. Each black dot reflects a genome-wide IR-raising genetic variant. The blue lines indicate regression and 95% CIs of IR on colorectal cancer risk (HR = 0.96, 95% CI: 0.78–1.17). CI, confidence interval; HR, hazard ratio; IR, insulin resistance.

In addition, we conducted MR analyses in the subgroups stratified by BMI, physical activity, and dietary fat intake (Figure 1). In both physical activity-stratified subgroups (i.e., MET ≥ 10 and <10), genetically elevated IR was associated with a reduced risk for CRC. Similarly, in the high-fat diet subgroup, genetically raised IR was associated with decreased CRC risk. However, in the low-fat diet (calories from SFA <7%) subgroup, genetically raised IR was associated with increased risk for CRC, although the effect in this low-fat group was not statistically significant. We further performed an overall pooled MR analysis by combining all the IR SNPs (Figure 2) and found no evidence of statistically significant association between genetically predicted IR and CRC risk (HR = 0.96, 95% CI: 0.78–1.17).

We also conducted a sensitivity test for the effect of IR genetic-instrumental variables on the risk for CRC by replacing current HRs with HRs obtained from the regressions adjusted for age and the 10 genetic PCs only. Similar MR results were observed, and no apparent directional pleiotropy was observed.

## Discussion

We evaluated the genetically determined effect of IR phenotypes on the risk for CRC in postmenopausal women by conducting an MR analysis, which could improve causal inference. If the MR study is not affected by pleiotropic effects through any alternative pathway, it could provide as robust a causal inference as randomized clinical trials do (19). Of note, our study included nonoverlapping genetic variants between IR and obesity, indicating the exclusion of potential pleiotropic effect from obesity. In addition, the MR approach could exhibit the lifelong effect of exposure (IR) on CRC risk, and compared with an observational study, its results are less susceptible to reverse causality. Thus, although our MR study was not designed to explore directly biological mechanisms, our findings suggest that the long-standing effect of IR is likely to affect the risk of postmenopausal CRC.

In particular, in our combined MR analysis by phenotype, genetically raised FG and FI exhibited their effects on the reduced risk for CRC, but with a lack of statistical power. Previous observational studies reported no associations of FG and FI levels with CRC risk (17, 18). That may indicate that FG and FI reflect the glycogenolysis activity in relation to insulin sensitivity in the liver (30), thus perhaps representing a relatively short-term phenomenon of IR. In contrast, 2-h glucose levels reflect beta cell function and insulin sensitivity in skeletal muscle (31), which may represent relatively long-term exposure to IR. In addition, glycated hemoglobin, a form of hemoglobin with an attached glucose molecule after exposure to high glucose levels (32), and C-peptide, a molar secretion from beta cells, which is on an equal basis with insulin, may function as integrated indicators of more stable measures of IR (33). Thus, future research into those long-term biomarkers with CRC risk is warranted.

In our individual MR analysis, *G6PC2* rs560887 was the main contributor to the MR effect estimate of FG on reduced CRC risk. *G6PC2* opposes the action of glucokinase in beta cells and thus regulates glycolytic flux and glucose-stimulated insulin secretion (34). This genetic polymorphism can cause mild hyperglycemia from birth onward and ultimately the development of type 2 diabetes in adulthood. Thus, early detection of the prediabetic condition may lead to the treatment of other cancer risk factors such as hypercholesterolemia, rendering additional protection from CRC later in life (35). Further, this *G6PC2* genetic instrument and its association with CRC would have been missed without the incorporation of fatty acids (i.e., observed in the high-fat diet group). This line of inquiry calls for future biological function research.

Additionally, our genetic instrumental analyses for the individual FI-related SNPs indicated that rs722025 in the *MTRR* gene was a significant contributor to the genetically derived effect of FI on CRC risk. This association was observed only in the physically inactive subgroup. A genetic polymorphism in *MTRR* in adipose tissue may prompt endoplasmic reticular stress, leading to inhibited insulin signaling, and thus resulting in IR and type 2 diabetes (36). In previous studies, this genetic polymorphism has been associated with several cancer types such as lung (37), stomach (38, 39), liver (40), and colorectal (41–44) cancers. Our findings of its relationship with IR and CRC are consistent with the findings of the aforementioned studies that reported negative (congruent direction with our finding) (41) or positive association with CRC risk (42–44), but our findings draw attention to the interaction with obesity factors because the association between the SNP and CRC risk was detected in physically inactive women only. This suggests that the analysis integrated with obesity-related lifestyle factors is essential. The SNP's protective effect on CRC risk in the inactive subgroup may be due to unmeasured confounders or other epigenetic pathways; thus, further molecular biologic–mechanism studies were needed to confirm our result.

*MKLN1* is an intracellular protein that mediates cell responses to the extracellular matrix and that influences cell adhesion and cytoskeleton organization (45, 46). It has been known to be associated with pancreatic (46) and lung cancer (47) and is a novel marker for cardiovascular risk (48). It has also been associated with type 2 diabetes (49). Our findings of its association with IR phenotypes are consistent with previous results, but our study newly reported the association of *MKLN1* with CRC risk. This association would have been missed without the incorporation of the physical activity factor, which will further require additional studies on larger populations to clarify the possible role of variation in this gene in colorectal carcinogenesis.

Our MR study results should be interpreted with some caution because of several assumptions required to be met. First, SNP instruments may not be correlated with other SNPs. We properly addressed this issue by pruning correlated SNPs. Second, the genetic variants must explain substantially the respective phenotypes. We included those SNPs having a strong association signal with their related phenotype. For the confounding factors that could introduce bias, we reduced the pleiotropic effect from obesity by using the following methods: (1) in our earlier GWA G × E study for the association of IR, we performed stratification analyses by obesity and associated lifestyle factors, which could have reduced the modifying effects of such factors before this MR analysis, and (2) in the current study, we estimated HRs for CRC by adjusting for potential confounding factors such as lifestyle and reproductive factors as well as 10 genetic PCs and further examined the association between genetically determined IR and CRC risk. Nonetheless, our results could be biased due to residual unmeasured confounding factors.

In addition, our pooled estimates by phenotype were not shown to be statistically significant in the overall analysis, but some significant associations in obesity subgroups were detected. This may reflect the heterogeneity of individual SNPs' estimates in the overall causal pathway connected to CRC risk and suggest the potential existence of genetically determined IR-outcome association that interacts with obesity factors. Obesity may act upstream of IR; that is, the effect of obesity between IR and CRC is substantial, so removing obesity could yield less reliable MR estimates (i.e., a weak direct effect of IR on CRC risk). Further, our results may indicate that biological pathways other than IR exist between obesity and CRC development. Last, we decreased the potential for population structure bias by adjusting for the population correction between IR and CRC in this individual-level analysis of the exposure and outcome data obtained from the same population.

MR results may also be subject to a nonlinear relationship between exposure and outcome. In our study, the genetically determined IR and its association with CRC risk may have been affected by a feedback mechanism (e.g., canalization), resulting in nonlinearity. But such canalization tends to bias MR results toward the null, so altering the statistical directions or significance is less likely (50). Our study may overfit the analysis owing to the nature of the data, where the exposure and outcome were obtained from the same study population. Finally, we examined 13 identified genetic instruments, and the results could have inflated false-positive rates due to multiple comparisons.

In summary, we attempted to improve the causal inference between IR and CRC risk and quantified the association by using genetic instruments in an MR framework. We obtained complex evidence that lifetime exposure to IR is likely to influence the risk for CRC in postmenopausal women. Future metabolic biologic study of this complicated association between IR and CRC by incorporating behavioral factors is warranted to clarify the underlying mechanisms of the associations we observed. Nonetheless, our results may contribute to building additional evidence for promoting intervention trials to lower IR and thus, to reduce CRC risk.

## Data Availability Statement

The datasets presented in this article are not readily available because the data that support the findings of this study are available in accordance with policies developed by the NHLBI and WHI in order to protect sensitive participant information and approved by the Fred Hutchinson Cancer Research Center, which currently serves as the IRB of record for the WHI. Requests to access the datasets should be directed to the corresponding author.

## Ethics Statement

The studies involving human participants were reviewed and approved by the Institutional Review Boards of each participating clinical center of the WHI and the University of California, Los Angeles. The patients/participants provided their written informed consent to participate in this study.

## Author Contributions

SJ, JP, ES, and Z-FZ designed the study. SJ performed the genomic data QC. SJ, JP, and ES performed the statistical analysis and interpreted the data. ES and Z-FZ supervised the genomic data QC and analysis and participated in the study coordination and interpreting the data. SJ secured funding for this project. All participated in the paper writing and editing. All authors have read and approved the submission of the manuscript. All authors contributed to the article and approved the submitted version.

## Conflict of Interest

The authors declare that the research was conducted in the absence of any commercial or financial relationships that could be construed as a potential conflict of interest.
