# Supplementary material for: Mendelian Randomization Study: The Association Between Metabolic Pathways and Colorectal Cancer Risk
Source: Front Oncol. 2020 Jul 23;10:1005. doi: 10.3389/fonc.2020.01005 (PMC7396568; doi:10.3389/fonc.2020.01005)
Supplement: Supplementary file 1 [file Table_1.docx]

Table S1. Sum of squared T statistics

| **Phenotype** |  | **Number of SNPs by phenotype and subgroup** | | | | | | | | | | |  | **Sum of squared T, p-value** |
| --- | --- | --- | --- | --- | --- | --- | --- | --- | --- | --- | --- | --- | --- | --- |
|  |  | **Overall** |  | **BMI1** |  | **PA0** |  | **PA1** |  | **SFA0** |  | **SFA1** |  |  |
| **Fasting glucose** |  | 1 |  |  |  | 2 |  |  |  |  |  | 1 |  | 129.1, P < 0.05 |
| **Fasting insulin** |  |  |  | 1 |  |  |  | 1 |  | 1 |  |  |  | 91.7, P < 0.05 |
| **HOMA-IR** |  | 2 |  |  |  |  |  |  |  | 2 |  | 2 |  | 126.1, P < 0.05 |
|  |  |  |  |  |  |  |  |  |  |  |  |  |  |  |
| **Sum of squared T, p-value** |  | 100.1, P < 0.05 |  | 31.2, P < 0.05 |  | 60.1, P < 0.05 |  | 30.7, P < 0.05 |  | 90.3, P < 0.05 |  | 100.4, P < 0.05 |  | 346.8, P < 0.05 |

BMI, body mass index; HOMA-IR, homeostatic model assessment–insulin resistance; PA physical activity, SFA, saturated fatty acids; SNP, single–nucleotide polymorphism.

Note: BMI1, BMI ≥ 30; PA0, active group (metabolic equivalent [MET] ≥ 10); PA1, inactive group (MET < 10); SFA0, % calories from SFA < 7.0; SFA1, % calories from SFA ≥ 7.0.
