# Supplementary material for: Mendelian Randomization Study: The Association Between Metabolic Pathways and Colorectal Cancer Risk
Source: Front Oncol. 2020 Jul 23;10:1005. doi: 10.3389/fonc.2020.01005 (PMC7396568; doi:10.3389/fonc.2020.01005)
Supplement: Supplementary file 2 [file Table_2.docx]

Table S2. Mendelian randomization-Egger test results*

|  |  | **Number of SNPs by phenotype and subgroup** | | | | | | | | | | | | |  |  |
| --- | --- | --- | --- | --- | --- | --- | --- | --- | --- | --- | --- | --- | --- | --- | --- | --- |
| **Phenotype** |  | **Overall** |  | **BMI1** |  | **PA0** |  | **PA1** |  | **SFA0** |  | **SFA1** |  | **Pooled RR (95% CI), p-value** | | |
| **Fasting glucose** |  | 1 |  |  |  | 2 |  |  |  |  |  | 1 |  | 0.97 (0.76 – 1.24), P = 0.641 | | |
| **Fasting insulin** |  |  |  | 1 |  |  |  | 1 |  | 1 |  |  |  | 0.46 (0.13 – 1.63), P = 0.081 | | |
| **HOMA-IR** |  | 2 |  |  |  |  |  |  |  | 2 |  | 2 |  | 0.23 (0.01 – 9.29), P = 0.230 | | |
|  |  |  |  |  |  |  |  |  |  |  |  |  |  |  | | |
| **Pooled RR (95% CI),**  **p-value** |  | 1.06 (0.70 – 1.61),  P = 0.335 |  | N/A |  | N/A |  | N/A |  | 0.50 (8.4e-12 - >20),  P = 0.783 |  | 0.92 (0.75 – 1.12),  P = 0.114 |  | 0.95 (0.83 – 1.09),  P = 0.409 | | |

BMI, body mass index; CI, confidence interval; HOMA-IR, homeostatic model assessment–insulin resistance; PA physical activity; RR, risk ratio; SFA, saturated fatty acids; SNP, single–nucleotide polymorphism.

Note: BMI1, BMI ≥ 30; PA0, active group (metabolic equivalent [MET] ≥ 10); PA1, inactive group (MET < 10); SFA0, % calories from SFA < 7.0; SFA1, % calories from SFA ≥ 7.0.

* MR-Egger test cannot estimate SE with single or two SNPs.
